# Home discharge and management in severe DBP patient

**DOI:** 10.1186/1824-7288-41-S1-A11

**Published:** 2015-09-24

**Authors:** Patrizio Fiorini

**Affiliations:** 1 Neonatal Intensive Care AOU Meyer Firenze, Italy

## 

Bronchopumonary dysplasia( DBP) is the most common sequelae related to very low birth weight, has a multifactorial aetiology and first characterized by Northway(1967), now unlike the original form of the disease, new form often develops in preterm newborns who may have needed little or no ventilator support, and have had low inspired oxygen concentration during early postnatal days. “New” DBP affects newborn with gestational age very low, could be the result of impaired lung growth (impaired alveolar or vascular growth). Affects about 68% of newborn between 22 and 26 gestational weeks and about 25% of newborn weight >1500gr. New DBP is defined as oxygen dependence for at least 28 days after birth, depending on the degree of respiratory support at 36 weeks of postnatal age or at the discharge, the disease is classified as mild, moderate or severe. Home discharge of patient with severe DBP is considered if the following criteria are fulfilled: a- sustained weight gain for a long time; b-maintains a normal body temperature fully clothed in an open bed; c- shows competent suckle feeding without cardiorespiratory compromise; d- has not had a relevant apnea, bradycardia, or oxygen desaturation for at least five days prior to discharge; e- demonstration of parental competence in all aspect of infant care; f- parental participation in a postnatal care program. Prior to discharge the parents are taught home resuscitation on the neonatal unit. They also receive a visit from the home oxygen nurse who discusses and gives training to the parents in the use of home oxygen. The equipment for domiciliary oxygen is a liquid oxygen system (pressurize gas cylinders, concentrators) comprising a storage vessel and a small portable vessel which can easily filled. The oxygen is supplied with a low-flow-meter via mono or binasal canula. A pulse oxymeter is prescribed to monitor SaO2 and cardiac frequency. Optimal SaO2 targets have to be further investigated by controlled studies but actually is > 93%. One the most challenging aspects of the treatment of DBP is the management of ventilator assisted children and tracheostomy in the home. Caregivers are trained in emergency procedures including CPR, tracheostomy changes and manual ventilator. Maintenance of oxygenation and proper nutritional support are critical aspects in the post-discharge management of these infants as immunization and neurodevelopmental follow-up.
